# Supplementary material for: Resolving the full spectrum of human genome variation using Linked-Reads
Source: Genome Res. 2019 Apr;29(4):635–45. doi: 10.1101/gr.234443.118 (PMC6442396; doi:10.1101/gr.234443.118)
Supplement: Supplemental Material [file supp_gr.234443.118_Supplemental_longranger-2.2.zip › longranger-2.2.tar.bz2!/lr2.2-public/lib/stan/cmdstan/stan/doc/api/html/classstan_1_1gm_1_1function__signatures-members.html]

Stan: Member List


|  |  |
| --- | --- |
|  | Stan  2.5.0  probability, sampling & optimization |


- Main Page
- Related Pages
- Namespaces
- Classes
- Files

- Class List
- Class Index
- Class Hierarchy
- Class Members

All Classes Namespaces Files Functions Variables Typedefs Enumerations Enumerator Friends Macros Pages

- stan
- gm
- function\_signatures

stan::gm::function\_signatures Member List

This is the complete list of members for stan::gm::function\_signatures, including all inherited members.

|  |  |  |
| --- | --- | --- |
| add(const std::string &name, const expr\_type &result\_type, const std::vector< expr\_type > &arg\_types) | stan::gm::function\_signatures |  |
| add(const std::string &name, const expr\_type &result\_type) | stan::gm::function\_signatures |  |
| add(const std::string &name, const expr\_type &result\_type, const expr\_type &arg\_type) | stan::gm::function\_signatures |  |
| add(const std::string &name, const expr\_type &result\_type, const expr\_type &arg\_type1, const expr\_type &arg\_type2) | stan::gm::function\_signatures |  |
| add(const std::string &name, const expr\_type &result\_type, const expr\_type &arg\_type1, const expr\_type &arg\_type2, const expr\_type &arg\_type3) | stan::gm::function\_signatures |  |
| add(const std::string &name, const expr\_type &result\_type, const expr\_type &arg\_type1, const expr\_type &arg\_type2, const expr\_type &arg\_type3, const expr\_type &arg\_type4) | stan::gm::function\_signatures |  |
| add(const std::string &name, const expr\_type &result\_type, const expr\_type &arg\_type1, const expr\_type &arg\_type2, const expr\_type &arg\_type3, const expr\_type &arg\_type4, const expr\_type &arg\_type5) | stan::gm::function\_signatures |  |
| add(const std::string &name, const expr\_type &result\_type, const expr\_type &arg\_type1, const expr\_type &arg\_type2, const expr\_type &arg\_type3, const expr\_type &arg\_type4, const expr\_type &arg\_type5, const expr\_type &arg\_type6) | stan::gm::function\_signatures |  |
| add(const std::string &name, const expr\_type &result\_type, const expr\_type &arg\_type1, const expr\_type &arg\_type2, const expr\_type &arg\_type3, const expr\_type &arg\_type4, const expr\_type &arg\_type5, const expr\_type &arg\_type6, const expr\_type &arg\_type7) | stan::gm::function\_signatures |  |
| add\_binary(const ::std::string &name) | stan::gm::function\_signatures |  |
| add\_nullary(const ::std::string &name) | stan::gm::function\_signatures |  |
| add\_quaternary(const ::std::string &name) | stan::gm::function\_signatures |  |
| add\_ternary(const ::std::string &name) | stan::gm::function\_signatures |  |
| add\_unary(const ::std::string &name) | stan::gm::function\_signatures |  |
| get\_result\_type(const std::string &name, const std::vector< expr\_type > &args, std::ostream &error\_msgs) | stan::gm::function\_signatures |  |
| get\_signature\_matches(const std::string &name, const std::vector< expr\_type > &args, function\_signature\_t &signature) | stan::gm::function\_signatures |  |
| instance() | stan::gm::function\_signatures | static |
| is\_defined(const std::string &name, const function\_signature\_t &sig) | stan::gm::function\_signatures |  |
| is\_user\_defined(const std::pair< std::string, function\_signature\_t > &name\_sig) | stan::gm::function\_signatures |  |
| key\_set() const | stan::gm::function\_signatures |  |
| num\_promotions(const std::vector< expr\_type > &call\_args, const std::vector< expr\_type > &sig\_args) | stan::gm::function\_signatures |  |
| reset\_sigs() | stan::gm::function\_signatures | static |
| set\_user\_defined(const std::pair< std::string, function\_signature\_t > &name\_sig) | stan::gm::function\_signatures |  |


---

[ Stan Home Page ]

*© 2011–2014,
Stan Development Team.*
